# Lived experiences of Turkish community pharmacists toward person-centric care: a qualitative analysis

**DOI:** 10.1080/20523211.2023.2294942

**Published:** 2023-12-19

**Authors:** Elif Ulutas Deniz, Muhammad Kamran Rasheed, Rumeysa Eren, Hatice Gözeler

**Affiliations:** a Department of Pharmacy Management, Faculty of Pharmacy, Atatürk University, Yakutiye-Erzurum, Türkiye; b Department of Pharmacy Practice, College of Pharmacy, Qassim University, Saudi Arabia; c Faculty of Pharmacy, Atatürk University, Yakutiye-Erzurum, Türkiye

**Keywords:** Person-centric care, community pharmacist, public health, pharmacy

## Abstract

**Background:**

In Turkey, the SMART pharmacist program was launched to enable community pharmacists to deliver patient-centric care services. However, how far this programme has achieved success and what are the challenges faced by the programme need to be evaluated.

**Objective:**

This study aimed to explore the experiences of community pharmacists in providing person-centric care services in pharmacies and provide recommendations for improved care effectiveness.

**Methods:**

A phenomenological approach was adopted in this study. To conduct qualitative interviews, a semi-structured interview guide was devised to gather insights from the pharmacists. The interviews were coded verbatim. Subsequently, various themes and sub-themes were developed based on the aim and objectives of the study. A total of 14 pharmacists engaged in semi-structured interviews conducted between May and August 2023.

**Results:**

Two main themes emerged from the data: challenges in person-centric care and recommendations to improve person-centric care. Barriers were subdivided into patient-related challenges and personal challenges. Recommendations consisted of pharmacists’ professional tasks and recommendations.

**Conclusion:**

The findings of this study, suggest that the concerned healthcare authorities should re-evaluate the ‘SMART pharmacist’ program in Turkey and address professional and personal challenges faced by community pharmacists in delivering effective person-centric care. Pharmacists recommend patient follow-up (follow-ups), providing a counselling environment, collaboration with physicians, expansion of pharmacy services.

## IMPACTS ON PRACTICE

Raising awareness among pharmacists can be achieved through initiatives like the ‘SMART Pharmacist Program’, aimed at enhancing the provision of person-centric pharmaceutical care to patients.

Government policies may be required revision to community pharmacists greater authority.

Academic institutions could contemplate revising the existing pharmacy education curriculum to emphasise person-centric pharmacy education.

Likewise, professional organisations may need to reevaluate the continuing professional development prerequisites for community pharmacists.

## Introduction

Person-centric care, an updated version of patient-centric care, emphasises the importance of increased patient involvement in healthcare. It uses clinical skills, evidence-based knowledge, and the patient's perspective to provide personalised, coordinated care, empowering individuals to optimise their quality of life (Ekman et al., 2011; Lambert et al., 1997; Nolte et al., 2020; Sepp et al., 2022). The World Health Organisation emphasises the importance of person-centric health services, which involve recognising patients’ unique experiences, facilitating shared decision-making, promoting holistic care, engaging families, providing convenient access, facilitating navigation, creating a supportive environment, and enhancing staff communication skills (Da Costa et al., 2020).

Pharmacists are highly accessible healthcare professionals who provide free advice and direct patient medication requirements, significantly impacting drug therapy outcomes and patient’s quality of life (Nadem, 2023; Pirhan & Özçelikay, 2005; Sánchez, 2011). The pharmacy profession has shifted from a product-centric approach to a person-centric practice, with community pharmacists now providing patient-centric care services, making them ideal for enhancing public health due to their esteemed position (Anderson, 2002; Jackson et al., 2004). Community pharmacists are underutilised in person-centric healthcare. However, a recent shift in healthcare practices involving primary healthcare providers taking on a more active role as care extenders to address the appropriate use of medications, reduce the cost of therapy and improve patient access to healthcare (Mossialos et al., 2015).

Person-centric care practices in Turkey involve providing physician-prescribed medications, patient counselling, and pharmacist collaboration to optimise health and medication outcomes (Rabuş, 2016). Turkish Pharmacy legislation grants community pharmacists authority to dispensing medications to patients only, with no or limited provisions to medical procedures or measurements within the pharmacy setting (Ministry of Health, 2012). However, The SMART Pharmacist Program, launched in 2014 in Turkey aims to improve healthcare services for patients with conditions like diabetes, asthma, hypertension, chronic obstructive pulmonary disease, and dyslipidemia (Turkish Pharmacists Association, 2015). The SMART Pharmacist Program is a sustainable, evidence-based educational initiative that integrates all components of the Continuous Professional Development cycle, directly influencing daily pharmacy practice (Turkish Pharmacists Association, 2015). The Program, implemented by strong national leadership and key stakeholders, has empowered pharmacists to enhance professional competencies, expand services, improve service quality, and improve patient care outcomes (Rouse & Meštrović, 2020). Therefore, following The SMART program, this study emphasises the need to assess the personal experiences of pharmacists in Turkey to comprehend person-centric care services, identify potential improvements and to elucidate recommendations by pharmacists for the advancements of person-centric care in Turkey.

## Materials and methods

### Research design

The study was conducted in Erzurum, Turkey, involving community pharmacists at their workplace. The qualitative methodology was chosen for data collection to gain in-depth insights into community pharmacists’ experiences and perspectives on person-centric care, which could not be achieved with quantitative methods (Al-Busaidi, 2008; Pope et al., 2002). The qualitative methodology was adopted for this research to generate comprehensive data and aims to gain an understanding of a phenomenon, rather than confirming preconceived hypotheses in quantitative research (Chafe, 2017).

Phenomenology is a qualitative research method that centres on the exploration of an individual's lived experiences within the world (Williams, 2021). Given the educational background of the principal researcher and the keen interest of pharmacists in person-centric care, an interpretative phenomenological approach (IPA) was deemed more suitable and thus employed for this research work (Shaw & Anderson, 2018).

### Study participants and recruitment strategies

**Figure 1.**
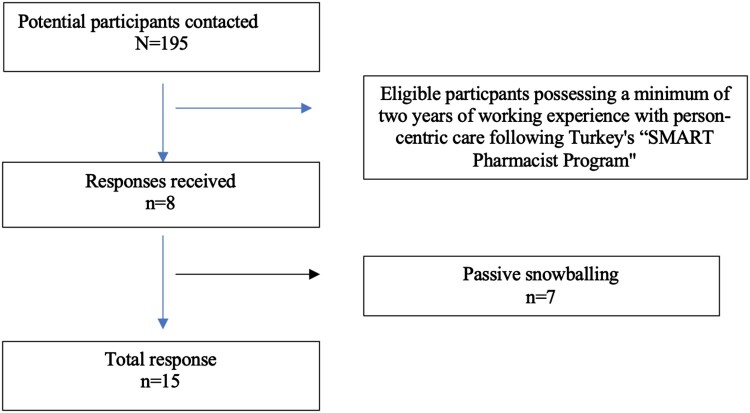
Flowchart of the participant recruitment process.

The recruitment process utilised a convenience-based purposive sampling strategy, focusing on community pharmacists within the city of Erzurum. The flowchart explaining the participant selection process was presented in Figure 1. To construct this purposive sample, invitations were extended to community pharmacists who possessed a minimum of two years of working experience with person-centric care following Turkey's ‘SMART Pharmacist Program’. Initially, an invitation message was disseminated through a WhatsApp group comprised of pharmacists registered with the relevant Pharmacist Chamber. Among the 195 community pharmacists registered with the Chamber of Pharmacists, 8 expressed a positive interest in participating. Subsequently, consenting participants were encouraged to refer colleagues who met the inclusion criteria to join the study. Following established practices in qualitative research with homogeneous subject groups, a target sample size of 10–15 participants was determined, guided by principles of data saturation (Mason, 2010). To facilitate the participation of interested individuals, a direct channel for contacting the research team was established. This facilitated the scheduling of interviews at mutually convenient times, dates, and locations. The participants weren't compensated for their time.

### Instrument development

To conduct qualitative interviews, a semi-structured interview guide was meticulously devised to gather insights from the community pharmacists. Semi-structured interviews were chosen as the preferred method for acquiring qualitative data due to their capacity to elicit in-depth descriptions of the interviewee's experiences. The development of the interview guide followed a comprehensive literature review (Rasheed et al., 2020 Sutton & Austin, 2015;) and subsequent discussions and refinement by all the authors. The interview guide was sent to two professionals for review and their feedback was incorporated to ensure its appropriateness and accuracy. The final iteration of the interview guide was piloted in two interviews at the commencement of this study (Gale et al., 2013).

### Data collection process

All the interviews were carried out in person at the workplace of community pharmacists. The interviews will be conducted till data saturation is reached, meaning that no new themes or insights will emerge from the interviews. Throughout both the data collection and subsequent analysis stages, the researcher maintained an attitude of reflexivity and self-scrutiny. It's important to note that neither the interviewer nor the participants had any prior acquaintance. At the outset of each interview, the principal investigator introduced herself, disclosed her profession as a pharmacist, provided details about her place of employment, and offered comprehensive information about the study. Prior to each interview, the principal researcher (EUD) presented the prospective participants with a brief overview of the study's goals and sought their consent for audio recording. Furthermore, participants were requested to sign a voluntary participation form. All interviews were audio-recorded using a high-quality recording device, the Sony ICD-PX470. All interviews were conducted in the Turkish language by the principal researcher (EUD), utilising a semi-structured interview guide (see Appendix) and lasted an average of 20 min (range: 10–30 min). During the interviews, no data that could potentially identify the respondents was collected. The foundation for data analysis relied solely on the transcripts of the recorded interviews. During the interviews, the interviewer made field notes, and additional notes were taken immediately following the interviews as necessary. These annotations helped ‘direct feedback’ from the participants, enabling immediate validation or dismissal of the central concepts discussed during the interviews.

In order to maintain participant anonymity, each participant will be denoted by the letter ‘P’ followed by their respective identification number (P 1-14). Participants were not subjected to any age, gender, or speciality restrictions. However, their demographic information was collected to present differentiation within the sample

### Data analysis

All audio recordings were transcribed verbatim, preserving the exact wordings of the participants. Subsequently, each transcript underwent a thorough review and correction process by the lead researcher (EUD). This review involved listening to the audio recordings while reading the transcripts to identify and rectify any spelling or other errors, ensuring a comprehensive understanding of the content. To protect the participants’ anonymity, the transcripts were anonymized to remove any information that could potentially identify them, such as names, places, or significant events. The transcriptions, originally in Turkish, were translated into English by a qualified English teacher. The English version was again translated back to the Turkish language by another professional, following a back-and-forth translation process to correct any inaccuracy of the recorded data. Following the transcription and translation processes, the implementation of interpretative phenomenological analysis (IPA) was undertaken as an integral component of the data analysis, with the overarching goal of attaining a deeper understanding of the experiences (Tuohy et al., 2013) conveyed by the survey participants. The analysis was facilitated using MaxQda 22.0.6 software. This analysis involved the collaboration of two researchers, employing researcher triangulation, to broaden the research perspective and validate the research findings (Carter et al., 2014). Themes and sub-themes were generated following the aim of the study project. Field notes, taken during the interviews, served as valuable supplementary information to support the overall process. This was particularly important to mitigate potential memory bias that could arise due to the time gap between the interviews and subsequent transcription and coding (Ritchie et al., 2013 Sutton & Austin, 2015;).

Quality criteria were followed and met in this qualitative research which include trustworthiness, credibility, transferability, dependability, and confirmability of the data (Guba, 1981):

#### Credibility

The primary data, which encompasses records and interview transcripts, is securely archived at Atatürk University Faculty of Pharmacy, ensuring accessibility for potential future inquiries. To bolster the robustness of the research process, an additional layer of quality assurance was integrated. During the initial interviews, a mutual cross-checking of interview recordings was conducted by both EUD and RE. This approach effectively mitigated the potential presence of leading questions, biases, or overlooked cues, thereby elevating the overall quality of the data collection process. The composition of the research team conforms to the criteria of a researcher triad, with two out of the three researchers being academic pharmacists in Turkey, while the third member is a pharmacy student actively engaged in Turkey.

#### Transferability

The manuscript furnishes an exhaustive account of the methodology, data collection, analysis, and interpretation, thus ensuring the replicability of the study across diverse contexts. These measures bolster the reliability and transferability of the research findings.

#### Dependability

To ensure the dependability of the findings, a rigorous approach was implemented. The results underwent independent analysis, and extensive discussions and comparisons took place among the three distinct investigators within the research team (EUD, HG, RE, MKR). This meticulous process significantly contributes to the reliability and robustness of the study's outcomes.

#### Confirmability

The reading and coding processes were independently conducted by the principal researchers (EUD and RE). Furthermore, the findings resulting from the thematic analysis were subjected to review, discussion, and validation by all experienced qualitative researchers involved in the study (EUD and MKR). A reflective review was carried out both before and after the analysis process to address potential biases that might influenced the analysis. This comprehensive approach greatly enhances the overall confirmability of the study's results.

### Ethical considerations

The research obtained approval from the Ataturk University Clinical Research Ethics Board (Approval No: B.30.2.ATA.0.01.00/439).

## Results

### Demographics of study participants

The point of data saturation was reached after completing 14 interviews, signifying the richness of the information gathered. In terms of gender distribution, the number of male and female participants was equal, aged between 30 and 34 years (57.2%), and had 6–10 years of community pharmacy experience (57.1%). Of the participants, 57% were recruited through purposive convenience sampling, while the remaining 43% were engaged via passive snowballing. Furthermore, participants exhibited a wide spectrum of ages and working experience, thereby enriching the demographic diversity. The demographic characteristics of the participants are comprehensively presented in Table 1.

**Table 1.** Demographic characteristics of study participants (*n* = 14).

Characteristics	Parameters	Frequency (%)
Gender	Male	7 (50)
	Female	7 (50)
Age (years)	25–29	2 (14.3)
	30–34	8 (57.2)
	35–39	3 (21.4)
	40–44	1 (7.1)
Years in practice (community pharmacy setting)	2–5	2 (14.3)
	6–10	8 (57.1)
	11 and above	4 (28.6)

Two themes and subsequent sub-themes emerged from the dataset (Table 2).

**Table 2.** Themes and sub-themes.

Themes	Sub-themes	Categories	Description
Challenges to provide person-centric care	Patient-related challenges	Purchasing over-the-counter drugs online	Purchasing of over-the-counter (OTC) medicines from online platforms
		Low trust in pharmacists	Perception of pharmacy as a business
		Social media influence	Relying on drug information on social media
	Personal challenges	Limited authority	The inability to perform many practices in the pharmacy
		Excessive management procedures	Paperwork to be followed in the pharmacy
Recommendations towards effective person-centric care	Pharmacists’ professional tasks	Offering comprehensive medication information for chronic patients	Detailed explanation about medicines
		Providing lifestyle recommendations	In addition to the diseases, extra lifestyle recommendations
		Recommending food supplements	Suggesting food supplements to aid treatment
		Patient follow-up	Monitoring the patient's treatment process
		Physician-pharmacist collaboration	Pharmacist-physician cooperation in the treatment management
		Magistral preparation	The supply of medicines other than ready-made medicines in the pharmacy
		Providing a counselling environment	The allocation of a special area in the pharmacy for counselling
		Lifelong learning	Continuous self-improvement of the pharmacist
		Effective communication	The pharmacist's effective communication skills
		Utilisation of technology	Effective use of technology by pharmacists
	Recommendations	The expansion of pharmacy-based services	Increasing the authorities of pharmacists
		Person-centred care-focused undergraduate education	Education prioritising person-centred care
		Clinical pharmacists	The involvement of clinical pharmacists in community pharmacies

### Theme 1: challenges to provide person-centric care

The challenges theme consists of two sub-themes: patient-related challenges like online drug purchases and low trust in pharmacists, and personal challenges like limited authority and financial constraints.

#### Subtheme 1.1: patient-related challenges

*Purchasing over-the-counter drugs online:* Some pharmacists stated that the online sale of OTC products is a serious public health concern. They noted the presence of counterfeit medicines in the online market and expressed regret that they cannot provide adequate counselling services on this issue, as patients often self-administer these products.

Many medicines are sold online, and they can have side effects. These medications are frequently counterfeit, and when patients use them, believing they are genuine, they assume they are self-treating. However, when they experience harm, they mistakenly attribute it to the legitimate medication they purchased from the pharmacy. It is crucial to prevent this, as it presents a significant danger. (P8)

*Low trust in pharmacists:* Most pharmacists stated that patients do not trust them as much as they trust their doctors. Consequently, pharmacists reported that they are sometimes unsuccessful in recommending medications to patients. One of the interviewed pharmacists is concerned that patients perceive them as more business-oriented.

As community pharmacists, we engage in communication with patients at a level similar to that of doctors. However, patients don't always fully recognize the depth of our expertise. Some may assume that we have commercial interests influencing our recommendations, which can create a barrier between us and the patients. (P10)

*Social media influence:* Some pharmacists reported that patients frequently acquired healthcare-related information from social media platforms without consulting or verifying either the pharmacists or physicians. They stated that it's often the case that patients seek professional advice only after experiencing harm from unverified healthcare-related information from social media. Furthermore, these pharmacists expressed concerns about the lack of regulatory oversight on social media, particularly in Turkey, where community pharmacists are prohibited from advertising medicines or related products according to pharmacy legislation.

Patients often turn to information they find on social media, sometimes leading to the adoption of incorrect practices. They may only seek our advice when their health condition has already worsened significantly. Nonetheless, we continue to make efforts to assist them to the best of our abilities. (P-11)

#### Subtheme 1.2: personal challenges

*Limited authority:* The majority of pharmacists indicated that their authority to conduct person-centric care within pharmacies is constrained.

Our current regulations restrict us to the tasks specifically designated for patient follow-up. For instance, we are unable to conduct blood sugar measurements for diabetic patients or monitor blood pressure for hypertensive patients. (P5)

*Excessive administrative responsibilities:* The majority of pharmacists mentioned that they grapple with an excessive amount of administrative work associated with pharmacy operations, which subsequently hampers their ability to dedicate sufficient time to person-centric care.

There is too much paperwork. There are too many procedures. A community pharmacist is struggling with incredible procedures in the background. (P9)

### Theme 2: Recommendations towards effective person-centric care

The second theme emphasises community pharmacists’ role in person-centric care, including medication information, lifestyle recommendations, alternative treatment options, patient follow-up, collaboration, lifelong learning, effective communication, technology utilisation, legal requirements, public awareness, pharmacy expansion, and undergraduate education.

#### Subtheme 2.1: pharmacists’ professional tasks

*Offering comprehensive medication information in chronic diseases*: All pharmacists agreed that they should give detailed information about the use of medication, potential side effects, any drug-nutrient or drug–drug interactions and how to achieve therapeutic outcomes with effective monitoring, especially in patients with chronic diseases.

Person-centric services are entirely dedicated to providing personalized treatment for each patient's unique medical conditions. For instance, when dealing with patients who have diabetes, asthma, or high blood pressure, pharmacists must offer recommendations that are tailored to each individual's needs. Additionally, pharmacists should inquire about other relevant health issues, such as whether the patient is taking cortisone, and provide guidance on potential side effects in consideration of the patient's overall health profile. (P13)

*Providing lifestyle recommendations*: Some of the pharmacists reported that it is important patient gets lifestyle modifications and non-pharmacological measures from community pharmacists for the prevention and management of diseases and to quality of life.

We should adopt a patient-centric approach in the pharmacy, tailoring our recommendations to each patient's unique profile. For instance, if a patient has high blood pressure, we should provide usage recommendations that are specific to their condition. Furthermore, we can suggest dietary changes, such as reducing salt and spicy foods in their diet. In the case of patients with asthma or COPD, we should advise them to avoid smoky environments and encourage spending more time in open areas. Similarly, for patients with diabetes, we should emphasize the importance of dietary considerations and offer guidance on managing their diet effectively. (P10)

*Recommending food supplements*: Most pharmacists noted that recommending food supplements alongside physician-prescribed medicines could improve patients’ health outcomes.

In my opinion, we can enhance the medical support provided to patients by incorporating elements such as vitamins or complementary therapies like herbal phytotherapy and aromatherapy. For instance, we could offer aromatherapy using various plants to support cancer patients, thereby improving their quality of life and potentially mitigating medication side effects. Pharmacists have the opportunity to further develop their capabilities in this regard. (P13)

*Patient follow-up:* Some pharmacists argued that patient follow-up should take place in community pharmacy settings in Turkey. They stated that it is important to monitor the patient’s progression toward achieving a therapeutic outcome.

If a patient experiences any issues after taking their medication, it is essential to reach out and invite them back to the pharmacy for further clarification. If there is any aspect of their treatment that they do not fully understand, we should encourage them to return or inquire about all of the patient's health conditions. (P13)

*Physician-pharmacist collaboration:* One of the pharmacists emphasised the importance of physician-pharmacist collaboration. He argued that the quality of life of the patient can be improved through mutual exchange of information.

I believe that the physician-pharmacist relationship should be further enhanced. Improved collaboration between physicians and pharmacists can lead to increased efficiency in patient care. Currently, doctors prescribe medication, and patients visit pharmacies to collect their prescriptions, often without much interaction. There may also be a lack of awareness among doctors regarding complementary therapies like phytotherapy and aromatherapy. I think it's essential to change this perspective and invest in our professional development in these areas. (P13)

*Magistral preparation:* Few pharmacists argued that pharmacies should be able to make magistral preparations, or even that pharmacists should have their standardised formulas. One pharmacist said that such preparations could be used for conditions such as bedsores in elderly patients.

Especially for inpatients, bedridden patients, patients with bed sores, physicians can go and look at them at home and apply treatment accordingly. You know, we can do this, we can help in patient treatment with our magistral medicines. (P4)

*Providing a patient counselling environment*: A limited number of pharmacists underscored the importance of establishing a distinct consultation space within the pharmacy, facilitating the provision of person-centric healthcare

The physical layout of the pharmacy is also of paramount importance. While an area of around 40–45 square meters may seem sufficient at present, I believe that it warrants some reconsideration. This is because it is crucial to establish a conducive and health-focused environment that allows for meaningful interactions between patients and pharmacists. (P12)

*Lifelong learning:* Some pharmacists have posited that the successful implementation of person-centric care in pharmacies necessitates a continuous commitment to self-improvement. They contend that pharmacists must enhance their knowledge and expertise, particularly in non-pharmaceutical products such as vitamins and dermocosmetics. They propose that professional associations and drug companies could offer both online and in-person training programmes to facilitate this ongoing development.

I believe that there is a significant need for us to enhance our skills in patient-oriented care services. We can strengthen these abilities through training, whether in-person or online. I say this because, in past years, it appears that previous generations of pharmacists may not have received specific training in patient-oriented care services. (P13)

*Effective communication*: Pharmacists underlined the critical importance of effective communication, specifically emphasizing the need to actively listen to patients and use language appropriate to the patient's level of understanding. They noted that personalised communication (formal/intimate) is a crucial factor affecting patient care.

Individualized communication with patients holds paramount significance. During one-on-one interactions, we may encounter patients with hearing difficulties, requiring us to speak more audibly. Patient preferences can vary widely, with some expecting a more heartfelt approach while others prefer a more formal one. It is crucial to assess each patient's expectations and adapt our communication style accordingly. (P14)

*Utilisation of technology*: Two pharmacists emphasised the importance of incorporating technology into pharmacy practices. They suggested that technology can serve various purposes, from streamlining pharmacy procedures to providing informative resources to patients.

We can offer patients additional information, not only within the pharmacy setting but also through online platforms and applications, especially. (P6)

#### Subtheme 2.2: recommendations

*The expansion of pharmacy-based services*: Nearly all pharmacists highlighted the potential for implementing person-centric services within pharmacies. They noted that within the constraints of pharmacy legislation, activities such as skin analysis, ear piercing, specific botox applications, low-risk injection procedures, and blood pressure and blood sugar measurements could be carried out in pharmacies.

‘I believe that skin analyses should become more widely available. Enhancing our analysis skills to offer tailored products and treatments to the right patients or customers is essential. I think this should be a standard service, accessible not only in select shopping malls and high street pharmacies.’ (P9)

‘Let's take Erzurum, for example. Services like blood sugar measurements and blood pressure monitoring have become highly sought-after in pharmacies. For instance, conducting regular blood pressure and diabetes sugar level checks can significantly boost patient loyalty to the pharmacy. As a result, we can establish a closer relationship with our patients.’ (P8)

*Person-centric care-focused undergraduate education*: One pharmacist recommended that revisiting their undergraduate training could be beneficial in enhancing person-centric care.

‘If our education in pharmacy faculty is primarily theory-focused, our approach to patient care in the pharmacy setting may need to adapt accordingly. In pharmacy education, the emphasis is often placed on the theoretical aspects of the field, and there may be limited exposure to practical, patient-centric experiences. During my time, and I'm not sure if this has changed in the current curriculum, we didn't receive extensive training on addressing the real-world clinical challenges patients face when using medications. Undergraduate education can be tailored to be more patient-centric.’ (P10)

*Clinical pharmacists*: A pharmacist argued that involving clinical pharmacists in the field would be advantageous, citing potential benefits for both patient care and employment opportunities.

‘I believe that a broader presence of clinical pharmacists, rather than community pharmacists, would be preferable. This expansion creates more employment opportunities and elevates the status of pharmacists within society.’ (P9)

## Discussion

This research endeavoured to investigate the practical application of person-centric care within community pharmacies, drawing upon the experiential knowledge of community pharmacists. The study discerned the barriers encountered by them in their pursuit of person-centric care and documented the recommendations put forth by them.

While pharmacists consistently rank at the top in public opinion surveys as one of the most trusted professions (Perepelkin, 2011), our study found that most pharmacists reported that patients do not trust them as much as physicians. They attributed this to society's limited knowledge about the capabilities of pharmacists. Similarly, in the existing literature, researchers assessing patients’ awareness of the non-traditional roles of community pharmacists have noted a lack of knowledge about pharmacists’ responsibilities (Tootelian et al., 2005). For example, a study in Canada investigating the awareness of pharmacists as diabetes specialists revealed that most patients with diabetes were unaware that pharmacists could specialise in diabetes care (Mansell & Perepelkin, 2011). Similarly, our study results showed that healthcare consumers viewed them as customers to pharmacists and there is a lack of acknowledgement to consider pharmacists as healthcare professionals. This observation aligns with a similar study, where most individuals visiting pharmacies identified themselves as customers (Perepelkin, 2011). The issue may stem from a lack of effective communication regarding the roles of pharmacists, including their own self-advocacy.

The interviewed pharmacists highlighted the potential health risks from social media influence on individuals seeking health-related information, thus reducing the need for healthcare professionals. They suggest collaboration with technology companies to label misinformation and experts to provide promotion and news content, addressing the viral nature of misinformation (Merchant et al., 2021).

The availability of medicines on the Internet has undoubtedly made purchasing more convenient for consumers. However, it has also resulted in a significant reduction or complete elimination of personal interactions between pharmacists and patients (Anderson, 2005; St George et al., 2004). Consequently, the public views them primarily as suppliers of pre-packaged medicines, contributing to a process of de-professionalization (Morton et al., 2015). The study found that some pharmacists are concerned about patients purchasing medicine-related products online, citing potential health risks and even advocating for its complete prohibition.

Furthermore, policymakers are increasingly concerned about the commercialisation of pharmacies, which could potentially undermine the professional framework of pharmacists, rather than promoting their integral role in the healthcare sector (Ilardo & Speciale, 2020; Traulsen & Almarsdóttir, 2005).

In our research, a significant number of pharmacists expressed dissatisfaction with their limited authority, noting their inability to deliver effective person-centric care to patients. Similarly, a study by Samancı-Tekin found a limited impact of pharmacists in public health initiatives in Turkey (Samancı Tekin, 2020).

The study results indicate that pharmacists in Turkey prioritise patient care, but they find limited time due to operational demands and excessive administrative responsibilities. These results can be compared with another study, where pharmacists face challenges in managing their professional workload, with official procedures (Çalgan et al., 2008).

Pharmacists are recognised as pivotal contributors to the judicious utilisation of pharmaceuticals, notably in the context of chronic disease management (Sancar, 2016). According to our findings, community pharmacists indicated that they should prioritise person-centric care, particularly in the context of chronic diseases.

Food supplements are mainly used for minor health issues, but people often lack trust in their effectiveness unless recommended by a healthcare professional (Es-Safi et al., 2020). The study suggests that community pharmacists should recommend food supplements as adjunctive products for managing chronic conditions, in addition to prescribed medications.

The study suggests that community pharmacists should provide person-centric care, including lifestyle recommendations like smoking cessation, weight management and exercise advice for conditions like cardiovascular diseases and asthma (Hassali et al., 2018; Rosenthal et al., 2018). However, it has also been reported that patients often perceive pharmacists as ‘medical professionals’ rather than wellness experts, which may affect their willingness to engage in discussions about healthy lifestyle choices (Blebil et al., 2022).

With the shift of drug production to the industry, the role of drug manufacturing has diminished, and patient-centric interventions have gained prominence. Nonetheless, it has been suggested that pharmacists can incorporate traditional methods while remaining open to innovations (Azzopardi, 2000; Tarhan & Şar, 2021). In our study, some pharmacists emphasised that, for patient-centric care, the importance of magistral drugs was highlighted.

Our study results showed that pharmacists preferred adding courses in pharmacy education that specifically focus on person-centric care. Similarly, one study in Turkey, emphasised the significance of incorporating courses into the pharmacy curriculum in Turkey, to effectively offer services like injections, blood sugar and blood pressure measurements and vaccination in pharmacies (Kızılay, 2022). A patient-centric model, where patients are experts in understanding their experiences, preferences, and disease perception, is proposed as more effective than the traditional pharmacist-centred model (Berger, 2009). In Turkey, the integration of clinical pharmacy, pharmaceutical care, pharmacotherapy, and rational drug use courses into the pharmacy curriculum has enhanced professional skills and supported patient-centric continuing education (Ekman et al., 2011).

The study results showed that better physician-pharmacist collaboration in Turkey would improve the treatment outcomes of patients. Including a pharmacist in healthcare teams has been linked to clinical and economic benefits in chronic disease states like hypertension, diabetes, and heart failure (Bennett et al., 2011 Santschi et al., 2012;). A qualitative study in Portugal found that pharmacists emphasise the need for community microbial resistance studies and stakeholder discussions to enhance antibiotic use (Roque et al., 2013).

The study results revealed that person-centric care goals could be achieved through patient follow-up by pharmacists. These findings are supported by a systematic review, where the benefits of pharmacist follow-up in enhancing patients’ blood pressure control and medication adherence to antihypertensive medications have been established (Reeves et al., 2021). In addition, a study in Turkey found that community pharmacists successfully resolved asthma and COPD-related healthcare issues among patients through interventions (Apikoglu-Rabus et al., 2016).

In our study, some pharmacists emphasised the importance of continuous self-improvement and highlighted the significance of participating in professional training programmes to deliver person-centric care. The Turkish Pharmacists Association's in-professional training programmes are underutilised, highlighting the need for improved coverage and effectiveness. Internet-based interactive techniques should be used to overcome spatial and temporal constraints. Some participants suggested mandating these training programmes as a crucial step for the continuous development of pharmacists (Kıran, 2015). Another proposal suggests the implementation of a continuous professional development crediting system for pharmacists, which is expected to help them stay updated and adapt to changing needs (Kızılay, 2022). A 2022 qualitative study found that while pharmacists generally appreciated on-the-job training programmes, they faced challenges like transportation, access, and financial barriers (Cerbin-Koczorowska et al., 2022).

The ubiquitous utilisation of computers, digital mobile phones, and the internet is promising to augment healthcare delivery and facilitate a shift toward a more person-centric approach (Glare et al., 2023). In our study, pharmacists argued that the effective use of technology by both pharmacists and patients would improve person-centric care outcomes.

The study highlights the importance of effective communication between pharmacists and patients for providing person-centric care (Perepelkin, 2011). Effective patient-pharmacist communication is crucial for improving health outcomes, and pharmacists must develop competence in patient-centric communication, characterised by openness, active listening, and clear communication (Göçmen, 2020 King et al., 2021; Naughton, 2018; Scarabelin et al., 2019; Ulutaş et al., 2015;).

In our study, few pharmacists recommended the necessity of establishing a dedicated area within pharmacies where pharmacists can provide consultations, thus facilitating person-centric care

Community pharmacists are increasingly offering essential patient care services like emergency refills, dosage adjustments, prescription therapy, minor ailment prescribing, therapeutic substitution, laboratory test ordering, and injection administration (Mossialos et al., 2015). The current pharmacy legislation in Turkey restricts pharmacists’ practice to primarily dispensing medications (Ministry of Health, 2012). The study found that most pharmacists advocate for the inclusion of practices like blood pressure and injection administration within their pharmacies, as they receive patient requests for these services.

The study results showed that pharmacists in Turkey prefer the active involvement of clinical pharmacists in community pharmacies for person-centric care, assisting patients in adopting healthy behaviours through proactive assessment, lifestyle changes, and self-management support. Furthermore, it was underscored that by identifying patients’ risk factors and providing patient care services, early disease diagnosis and effective treatment methods can be implemented (Akyilmaz, 2023 Göçmen, 2020;).

## Limitations

This study has several limitations worth noting. Firstly, the use of purposive sampling might introduce some sampling bias, as it relies on individuals who have the time and willingness to participate. Also, since the data collector/interviewer was a pharmacist herself, there can be the risk of bias. Thirdly, the interview responses were translated back, and there is a possibility that some data loss might have occurred. Lastly, the social desirability could also influence the results.

## Conclusion

The study underscores the necessity to enhance person-centric care in community pharmacy settings in Turkey. The study identifies several personal and professional challenges pharmacists face in providing person-centric care. The study suggests that the Turkish government policies should revise regulatory amendments, re-evaluate the SMART Pharmacist Program and empower community pharmacists to deliver person-centred care. The study also recommends a thorough review of pharmacy operating procedures in Turkey to reduce pharmacist workload and enhance patient interaction opportunities. The study suggests that Turkey's Pharmacy Colleges should re-evaluate their curricula, emphasizing person-centric care, and professional organisations should update CPD requirements and resources for community pharmacists.

## Appendix

### Interview questions

1. What services are currently offered by community pharmacies in Turkey?
2. What are your opinions on the current role and practices of community pharmacists in providing services to consumers in Turkey?
3. From your perspective, how can community pharmacists assist consumers in managing their medications and health conditions?
4. According to your observations, which patient-centred services are preferred or commonly requested by consumers in community pharmacies?
5. In your view, how can patient-centred care services be effectively implemented in Turkish community pharmacies?
6. What types of services would you like to see in community pharmacies to better support healthcare consumers in the future?
7. What challenges do you anticipate community pharmacies might encounter when transitioning to patient-centred care services?
8. What barriers or issues exist in the practice of community pharmacy in Turkey regarding the implementation of patient-centred care services, both in the past and looking ahead?
